# A neoadjuvant therapy compatible prognostic staging for resected pancreatic ductal adenocarcinoma

**DOI:** 10.1186/s12885-023-11181-x

**Published:** 2023-08-23

**Authors:** Lingyu Zhu, Shuo Shen, Huan Wang, Guoxiao Zhang, Xiaoyi Yin, Xiaohan Shi, Suizhi Gao, Jiawei Han, Yiwei Ren, Jian Wang, Hui Jiang, Shiwei Guo, Gang Jin

**Affiliations:** 1 Department of Pancreatic Hepatobiliary Surgery, Changhai Hospital, Naval Medical University, NO. 168 Changhai Road, Yangpu District, Shanghai, China; 2 Department of Pathology, Changhai Hospital, Naval Medical University, NO. 168 Changhai Road, Yangpu District, Shanghai, China

**Keywords:** Neoadjuvant therapy, Upfront surgery, Staging, Prognosis, Survival, Grade, Pancreatic cancer, Pancreatic ductal adenocarcinoma

## Abstract

**Objective:**

To improve prediction, the AJCC staging system was revised to be consistent with upfront surgery (UFS) and neoadjuvant therapy (NAT) for PDAC.

**Background:**

The AJCC staging system was designed for patients who have had UFS for PDAC, and it has limited predictive power for patients receiving NAT.

**Methods:**

We examined 146 PDAC patients who had resection after NAT and 1771 who had UFS at Changhai Hospital between 2012 and 2021. The clinicopathological factors were identified using Cox proportional regression analysis, and the Neoadjuvant Therapy Compatible Prognostic (NATCP) staging was developed based on these variables. Validation was carried out in the prospective NAT cohort and the SEER database. The staging approach was compared to the AJCC staging system regarding predictive accuracy.

**Results:**

The NAT cohort’s multivariate analysis showed that tumor differentiation and the number of positive lymph nodes independently predicted OS. The NATCP staging simplified the AJCC stages, added tumor differentiation, and restaged the disease based on the Kaplan-Meier curve survival differences. The median OS for NATCP stages IA, IB, II, and III was 31.7 months, 25.0 months, and 15.8 months in the NAT cohort and 30.1 months, 22.8 months, 18.3 months, and 14.1 months in the UFS cohort. Compared to the AJCC staging method, the NATCP staging system performed better and was verified in the validation cohort.

**Conclusions:**

Regardless of the use of NAT, NATCP staging demonstrated greater predictive abilities than the existing AJCC staging approach for resected PDAC and may facilitate clinical decision-making based on accurate prediction of patients’ OS.

**Supplementary Information:**

The online version contains supplementary material available at 10.1186/s12885-023-11181-x.

## Introduction

Pancreatic ductal adenocarcinoma (PDAC) is an aggressive malignant tumor of the gastrointestinal tract with a 5-year survival rate of 11% and surgical resection is considered to be the only potential cure [1, 2]. However, only 15–20% of the patients have resectable tumors at the time of diagnosis [3]. As preoperative neoadjuvant therapy (NAT) is increasingly being used for treatment, patients with borderline resectable or locally advanced PDAC (BR/LA-PDAC) may achieve margin-negative resection and have similar overall survival compared to those with initially resectable diseases [4–6]. However, pathological evaluation became more challenging after NAT and the cancer staging system has not been updated in parallel.

The American Joint Committee on Cancer (AJCC) TNM staging system is the most commonly used staging system [7], and has shown good prognostic ability for resected PDAC patients in several validation cohorts [8–11]. However, the AJCC staging system for PDAC is based on patient populations who did not receive NAT [12], and has suboptimal performance in patients undergoing resection after NAT. Four-category T group failed to demonstrate a good prognostic ability, and no statistically significant difference in the overall survival (OS) between stages II and III [13]. A more simplified and practical prognostic staging system is needed to accurately estimate the survival probability for resected PDAC regardless of the use of NAT. To provide better distinction ability, better predictive and prognostic biomarkers of tumor pathology such as tumor differentiation, should be incorporated into the staging model [14].

In this study, we proposed a prognostic staging system for PDAC patients that is compatible with NAT and UFS. We compared the prognostic ability of the NATCP staging with that of the AJCC staging system, and assessed its accuracy in the validation cohort.

## Methods

### Study cohort

**Fig. 1:**
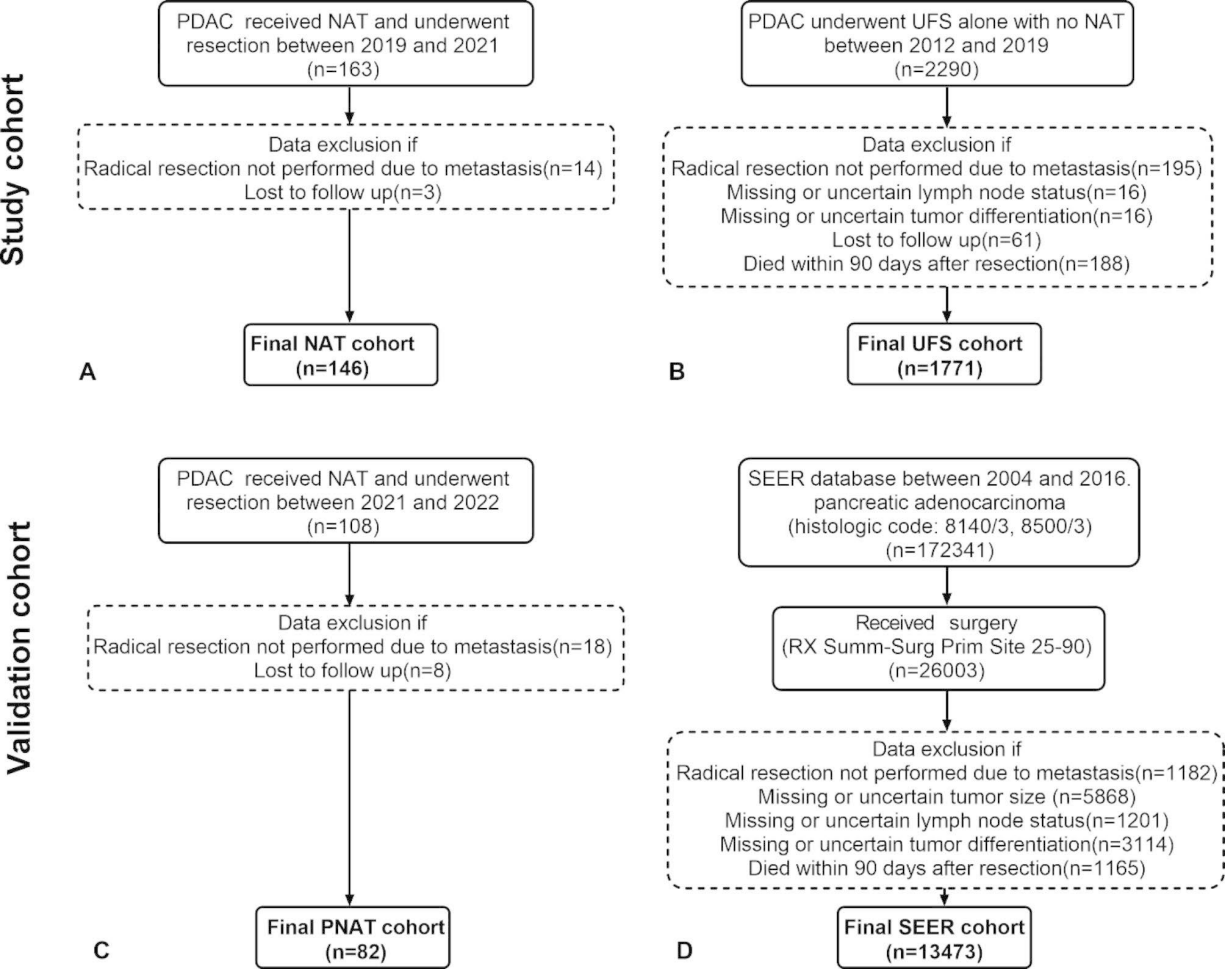
The patient selection criteria for the study cohort and validation cohort. PDAC, pancreatic ductal adenocarcinoma; NAT, neoadjuvant therapy; PNAT, prospective neoadjuvant therapy; UFS, upfront surgery; SEER, Surveillance, Epidemiology, and End, Results

The study protocol was approved by the Institutional Review Board of the Shanghai Changhai Hospital. In total, 146 consecutive patients with histologically confirmed PDAC, who received NAT and underwent resection between January 2019 and April 2021, were included in the NAT cohort (Fig. 1A). In addition, 1,771 consecutive patients who underwent UFS alone with no NAT between January 2012 and December 2019 were included in the UFS cohort (Fig. 1B). The two cohorts consisted of participants identified from a database that was prospectively maintained by the Department of Pancreatic Hepatobiliary Surgery of Changhai Hospital.

In our institution, the optimal treatment options were determined by the multidisciplinary team(MDT) based on the patients’ performance status, symptoms, tumor markers, and cross-sectional imaging. The initial assessment of pancreatic cancer was focused on surgical resectability. Based on the NCCN Clinical Practice Guidelines in Oncology, tumors were classified as resectable, borderline resectable (BR), locally advanced (LA) or metastatic, according to the degree of contact between the tumor and adjacent vessels/organs, and the presence of metastases. In general, the vast majority of resectable tumors underwent UFS, with NAT recommended as a priority for patients with a combination of high-risk factors including high carbohydrate antigen 19 − 9 (CA19-9) levels, large tumors, regional lymph nodes suspected metastases, significant weight loss, and pain. All BR/LA tumors were treated with NAT followed by resection, with some patients with specific genetic mutations having access to clinical trials[15]. Gemcitabine plus albumin-bound(nab) paclitaxel (AG) has been recommended as the standard first-line neoadjuvant treatment because we were conducting several relevant clinical trials with AG as the main focus. Other chemotherapy regimens includes gemcitabine plus tegafur/gimeracil/oteraci (GS), albumin-bound paclitaxel plus tegafur/gimeracil/oteracil (AS) and modified 5-fluorouracil, leucovorin, irinotecan, and oxaliplatin (mFOLFIRINOX). Surgical resection was commonly performed within four weeks from the last cycle of neoadjuvant chemotherapy.

The clinical data included demographics, body mass index, neoadjuvant chemotherapy regimen and cycle duration, CA19-9 level after neoadjuvant chemotherapy, and surgical information (such as tumor location, surgery type, and vascular resection). The pathological parameters, including tumor size, tumor differentiation, number of positive lymph nodes, and margin status, were recorded from the postoperative pathology reports. The Leeds Pathology Protocol (LEEPP) was routinely used for the pathological examination[16]. Margin status was defined according to the Royal College of Pathologists, with R0 corresponding to > 1 mm free margin and R1 corresponding to a tumor cell at 1 mm or less from the resection margin. Assessment of margin involvement comprised the superior mesenteric artery (SMA)/medial margin, proximal gastric or duodenal margin, distal jejunal margins, pancreatic neck margin, bile duct margin, the anterior surface, and the posterior margin [17–19]. Follow-up information was collected every 3 months by reviewing outpatient medical records or through phone interviews.

Patients who failed to undergo successful radical resection due to metastasis, died within 90 days after surgery, and were lost to follow-up were excluded from the analysis.

### Validation cohort

The validation cohort consisted of the prospective neoadjuvant therapy cohort (PNAT) cohort and the SEER cohort.We prospectively collected information on 82 patients who underwent pancreatectomy after NAT in our institution between May 2021 and March 2022 as a PNAT cohort (Fig. 1C). We followed up this cohort at three-month intervals and all patients were followed for more than one year after surgery.

PDAC patients who underwent upfront pancreatic surgery between 2004 and 2016 were selected from the SEER (Surveillance, Epidemiology, and End, Results) database and included in the SEER cohort. The patients had a confirmed primary cancer site in the pancreas (C25.0, C25.1, C25.2, C25.3, C25.7, C25.8, or C25.9) and ductal adenocarcinoma (SEER histological code: 8140/3, 8500/3). The detailed exclusion criteria of SEER are presented in Fig. 1D. Based on the eligibility criteria, 13,473 patients were included in the SEER cohort.

### Statistical analysis

Categorical variables are presented as absolute numbers with percentages referring to the corresponding group and were compared using Chi squared or Fisher’s exact test. Continuous variables are presented as the median and interquartile range (IQR). Parametric continuous variables are compared using the Student t test between cohorts, whereas non-parametric continuous variables are compared using the Mann-Whitney test. OS was calculated from the date of diagnosis to death or the last available follow-up. Median OS (mOS) was calculated using the Kaplan-Meier method and compared using log-rank test. Variables with p-value < 0.05 in the univariate analysis were incorporated into the multivariate Cox proportional hazard (PH) model. We set a p-value of > 0.1 as cutoff for removal to estimate the hazard ratios (HRs) for the corresponding clinicopathological features. The NATCP staging system restaged the disease according to the discriminative ability observed using the Kaplan–Meier curves for the NAT cohort. The Harrell concordance index (C index) was calculated using the bootstrap method to assess the predictive ability of the NATCP staging. Then, we evaluated the stratification ability of the NATCP staging in the UFS cohort and the validation cohorts. The receiver operating characteristic (ROC) curves of the NATCP and AJCC staging systems were compared in terms of survival at the specified time points (1year-, 2year-) in both NAT and UFS cohorts. The area under the curve (AUC) was calculated using logistic regression models.

The statistical analyses were performed using SPSS (version 22; IBM Corp., Armonk, NY, USA) and R (version 3.6.2; R Foundation for Statistical Computing, Vienna, Austria) software. P < 0.05 was considered statistically significant.

## Results

### Demographic and pathological characteristics

The NAT cohort included 146 consecutive patients with histologically confirmed PDAC who underwent resection after NAT between 2019 and 2021 at the Department of Pancreatic Hepatobiliary Surgery of Changhai Hospital. In total, 88 patients (60%) were males and 58 (40%) were females. The median age was 61 years [interquartile range (IQR): 54–45)] and the median body mass index was 22.66 kg/m[2] (IQR: 21.04–24.95). The majority of patients (77%) received a median of four (IQR: 4–6) cycles of AG regimen. In addition, 58 patients (40%) received stereotactic body radiotherapy before surgery. More than half of the patients (55%) achieved normalization of CA19-9 level after treatment. Approximately 62% of patients underwent pancreaticoduodenectomy, and 54 (37%) underwent additional vascular resection. After resection, 91% of patients received adjuvant therapy.

The histopathological analysis showed that 51 patients (35%) had tumors ≤ 2 cm in size, while 95 patients (65%) had tumors > 2 cm. Tumor differentiation was evaluated by experienced pathologists. There were 116 (78%) well-differentiated or moderately differentiated tumors and 30 (20%) poorly or undifferentiated tumors. Almost 47% of patients were lymph node-negative, while the remaining 41% were grouped as ypN1 and 12% as ypN2. According to the AJCC staging system, 55 (38%), 69 (47%), and 22 (15%) patients had stage I, II, and III tumors, respectively. R0 resection was achieved in 112 of the 146 cases (77%). Compared to the UFS cohort, patients in the NAT cohort were younger (p<0.001), had smaller tumor size after treatment (p<0.001), and were more likely to undergo vascular resection (p<0.001). In addition to these, patients received NAT were more inclined to continue adjuvant therapy postoperatively(p<0.001). The detailed patient information and tumor characteristics of the UFS cohort are presented in Table 1.

**Table 1:** Baseline and Pathological Data Between the NAT and UFS cohorts

	NAT Cohort	UFS Cohort	P value
	n=146(%)	n=1771(%)	
Sex			0.813
Male	88(60)	1085(61)	
Female	58(40)	686(39)	
Age,yr,(IQR)	61(54-65)	65(58-71)	<0.001
BMI,kg/m^2^,(IQR)	22.66(21.04-24.95)	22.55(20.60-24.48)	0.195
Tumor location			0.449
Head/Neck	90(62)	1147(65)	
Body/Tail	56(38)	624(35)	
Neoadjuvant Chemotherapy			
AG	112(77)		
GS	10(7)		
AS	18(12)		
mFFX	6(4)		
Cycle duration(IQR)	4(4-6)		
CA19-9(U/mL) after treatment			
≥37	65(45)		
<37	81(55)		
SBRT			
Yes	58(40)		
No	88(60)		
Type of surgery			0.288
PD	90(62)	1116(63)	
DP	48(33)	620(35)	
TP	8(5)	35(2)	
Vascular resection			<0.001
Yes	54(37)	145(8)	
No	92(63)	1626(92)	
Tumor size			<0.001
≤2 cm	51(35)	368(21)	
>2 cm	95(65)	1403(79)	
Tumor differentiation			0.785
Well-Moderate	116(78)	1390(78)	
Poor-Undifferentiation	30(22)	381(22)	
Number of positive lymph nodes			0.085
0	68(47)	945(53)	
1-3	60(41)	661(37)	
≥4	18(12)	165(10)	
Eighth AJCC-stage			0.257
I	55(38)	719(41)	
II	69(47)	848(48)	
III	22(15)	204(11)	
R-status			0.088
R0	112(77)	1240(70)	
R1	34(23)	531(30)	
Adjuvant therapy	133(91)	1257(71)	<0.001

NAT, neoadjuvant therapy; UFS, upfront sugery; IQR, interquartile range; BMI, body mass index; AG, albumin-bound paclitaxel plus gemcitabine; GS, gemcitabine plus tegafur/gimeracil/oteracil; AS, albumin-bound paclitaxel plus tegafur/gimeracil/oteracil; mFFX, modified 5-fluorouracil, leucovorin, irinotecan, and oxaliplatin; CA19-9, carbohydrate antigen 19-9; SBRT,Stereotactic body radiotherapy; PD, pancreatoduodenectomy; DP, distal pancreatectomy; TP, total pancreatectomy; AJCC, American Joint Committee on Cancer

### Univariate and multivariate predictors of survival

In the univariate analysis, there was a significant association between OS and tumor differentiation (HR, 2.604; 95% confidence interval [CI], 1.442–4.705; p = 0.002). Other clinicopathologic features include tumor size (≥ 2 cm; HR, 1.848, 95%CI, 1.029–3.319; p = 0.040), number of positive lymph nodes (p = 0.015; 1–3: HR, 2.046, 95%CI, 1.170–3.577; p = 0.012 and ≥ 4: HR, 2.526, 95%CI, 1.186–5.381; p = 0.016), and normalization of CA19-9 level after treatment (< 37 U/mL; HR, 0.558; 95%CI, 0.330–0.945; p = 0.030) were also observed to improve OS. These variables have been reported to be prognostic determinants in previous studies.

The clinical and pathological features with p-value < 0.05 on univariate analysis were entered into the multivariate Cox regression analysis. There was no collinearity of the analyzed variables. Only tumor differentiation (HR, 3.424; 95%CI, 0.521–7.712; p = 0.003) and the number of positive lymph nodes (p = 0.037; 1–3: HR, 1.835; 95%CI, 0.858–3.923; p = 0.117 and ≥ 4: HR, 3.366; 95%CI, 1.313–8.627; p = 0.011) (Table 2) were independently associated with OS.

**Table 2:** Univariate and Multivariate Cox Regression Analyses of Overall Survival for the NAT Cohort in Relation to Clinicopathological Features

	Univariate Cox Regression Analysis				Multivariate Cox Regression Analysis			
Characteristic	HR(95%CI)		P		HR(95%CI)		P	
Sex								
Male(reference)	─		─					
Female	1.063(0.637-1.772)		0.816					
Tumor location								
Head/Neck(reference)	─		─					
Body/Tail	0.635(0.368-1.094)		0.102					
Neoadjuvant Chemotherapy			0.686					
AG(reference)	─		─					
GS	1.631(0.692-3.843)		0.264					
AS	1.198(0.583-2.461)		0.622					
mFFX	0.846(0.205-3.495)		0.817					
CA19-9(U/mL) after treatment								
<37(reference)	─		─		─		─	
≥37	0.558(0.330-0.945)		0.030		1.385(0.803-2.387)		0.240	
SBRT								
Yes(reference)	─		─					
No	1.279(0.765-2.140)		0.348					
Type of Surgrey			0.147					
PD(reference)	─		─					
DP	0.562(0.316-1.001)		0.050					
TP	0.810(0.195-3.366)		0.771					
Vascular resection								
Yes(reference)	─		─					
No	0.628(0.380-1.039)		0.070					
Tumor size								
≤2 cm(reference)	─		─		─		─	
>2 cm	1.848(1.029-3.319)		0.040		1.298(0.661-2.551)		0.378	
Tumor differentiation								
Well-Moderate(reference)	─		─		─		─	
Poor-Undifferentiation	2.604(1.442-4.705)		0.002		3.424(1.521-7.712)		0.003	
Number of positive lymph nodes			0.015				0.037	
0(reference)	─		─		─		─	
1-3	2.046(1.170-3.577)		0.012		1.835(0.858-3.923)		0.117	
≥4	2.526(1.186-5.381)		0.016		3.366(1.313-8.627)		0.011	
Eighth AJCC-stage			0.046					
I(reference)	─		─					
II	2.071(1.137-3.770)		0.017					
III	2.088(0.944-4.621)		0.069					
R-stasus								
R0(reference)	─		─					
R1	0.756(0.401-1.422)		0.385					

NAT, neoadjuvant therapy; HR, hazard ratio; CI, confidence interval; P, p-value; AG, albumin-bound paclitaxel plus gemcitabine; GS, gemcitabine plus tegafur/gimeracil/oteracil; AS, albumin-bound paclitaxel plus tegafur/gimeracil/oteracil; mFFX, modified 5-fluorouracil, leucovorin, irinotecan, and oxaliplatin; CA19-9, carbohydrate antigen 19-9; SBRT,Stereotactic body radiotherapy; PD, pancreatoduodenectomy; DP, distal pancreatectomy; TP, total pancreatectomy; AJCC, American Joint Committee on Cancer

### Neoadjuvant therapy compatible prognostic staging

**Fig. 2:**
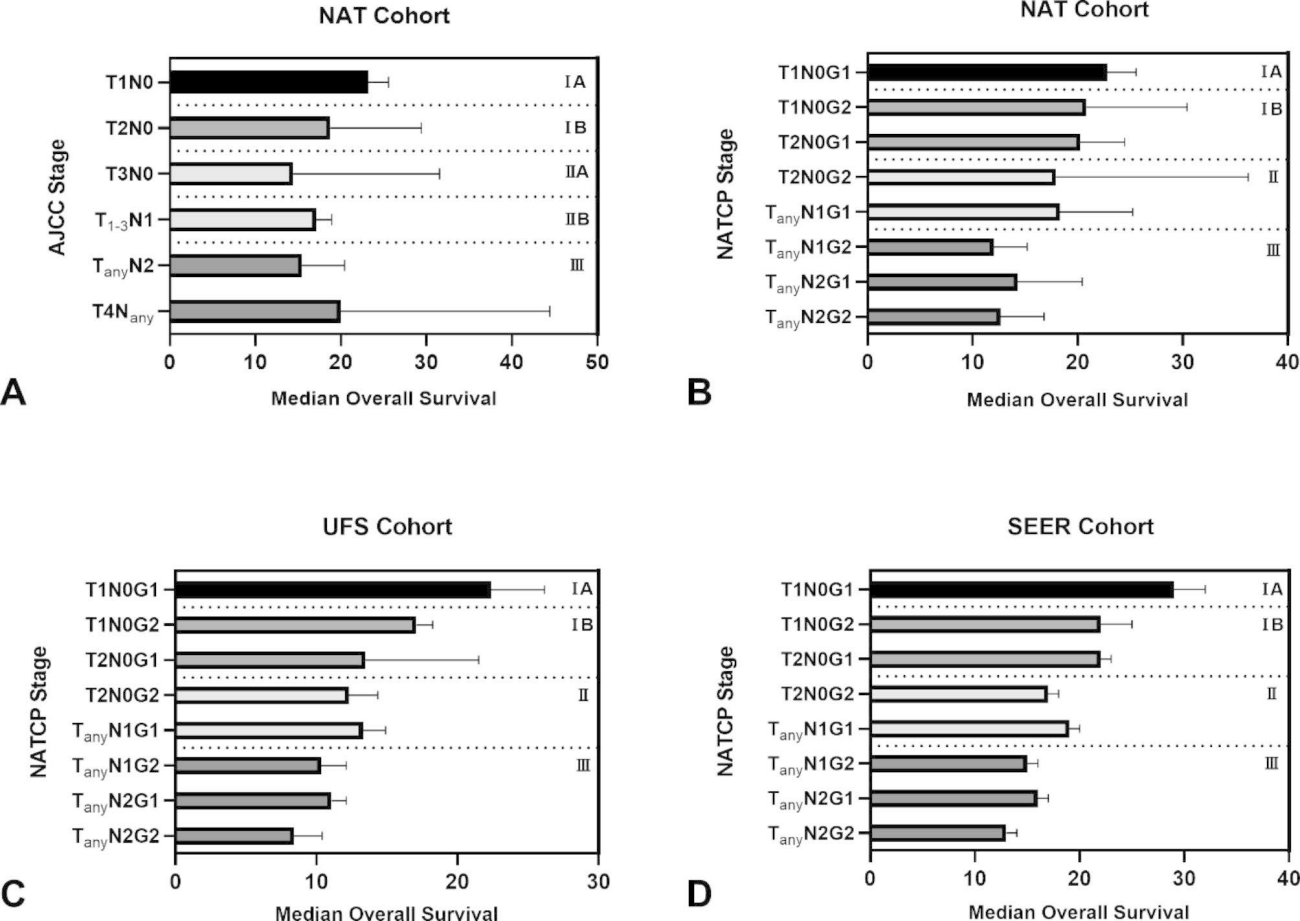
TNM stages and survival duration using the AJCC staging system for (**A**) the NAT cohort. NATCP stages and survival duration using the NATCP staging for (**B**) the NAT cohort, (**C**) the UFS cohort and (**D**) the SEER cohort. NAT, neoadjuvant therapy; UFS, upfront surgery; SEER, Surveillance, Epidemiology, and End, Results

Given the limited stratification ability of the T3 and T4 groups for overall survival in the NAT cohort (Fig. 2A), we modified the AJCC staging system by simplifying the T group, retaining the N group and adding tumor differentiation, which is an independent prognostic factor for OS. The NATCP staging was based on tumor size in two subgroups (T1: tumor ≤ 2 cm in the greatest dimension and T2: tumor>2 cm in the greatest dimension), lymph node status in three subgroups (N0: no regional lymph node metastasis, N1: metastasis in 1 to 3 regional lymph nodes and N2: metastasis in ≥ 4 regional lymph nodes) and tumor differentiation in two subgroups (G1: well/moderately differentiated and G2: poorly differentiated/undifferentiated) (Table 3). The NATCP stages were defined as stages IA (T1N0G1), IB (T1N0G2, T2N0G1), II (T2N0G2, TanyN1G1) and III (TanyN1G2, TanyN2G1, TanyN2G2) according to the Kaplan-Meier curves for the NAT cohort (Fig. 2B). A similar prognostic stratification trend could be seen in both the UFS cohort (Fig. 2C) and the SEER cohort (Fig. 2D).

**Table 3:** The Neoadjuvant Therapy Compatible Prognostic Staging Definitions and the 8th Edition of the AJCC Staging Definitions for PDAC

The 8th Edition of the AJCC Staging				The Neoadjuvant Therapy Compatible Prognostic Staging			
T1	Tumor ≤2 cm in the greatest dimension			T1	Tumor ≤2 cm in the greatest dimension		
T2	Tumor >2 cm and ≤4 cm in the greatest dimension			T2	Tumor >2 cm in the greatest dimension		
T3	Tumor >4 cm in the greatest dimension						
T4	Tumor involves CA, SMA, and/or CA, irrespective of size						
N0	No regional lymph node metastasis			N0	No regional lymph node metastasis		
N1	Metastasis in 1 to 3 regional lymph nodes			N1	Metastasis in 1 to 3 regional lymph nodes		
N2	Metastasis in ≥4 regional lymph nodes			N2	Metastasis in ≥4 regional lymph nodes		
				G1	Well/moderately differentiated		
				G2	Poorly differentiated/undifferentiated		
					G1		G2
T1N0		IA		T1N0	IA		IB
T2N0		IB		T2N0	IB		II
T3N0,T_1-3_N1		II		T_any_N1	II		III
T_any_N2, T4N_any_		III		T_any_N2	III		III

AJCC, American Joint Committee on Cancer; CA, celiac axis; SMA, superior mesenteric artery; HA, hepatic artery

### Performance in the study and validation cohorts

**Fig. 3:**
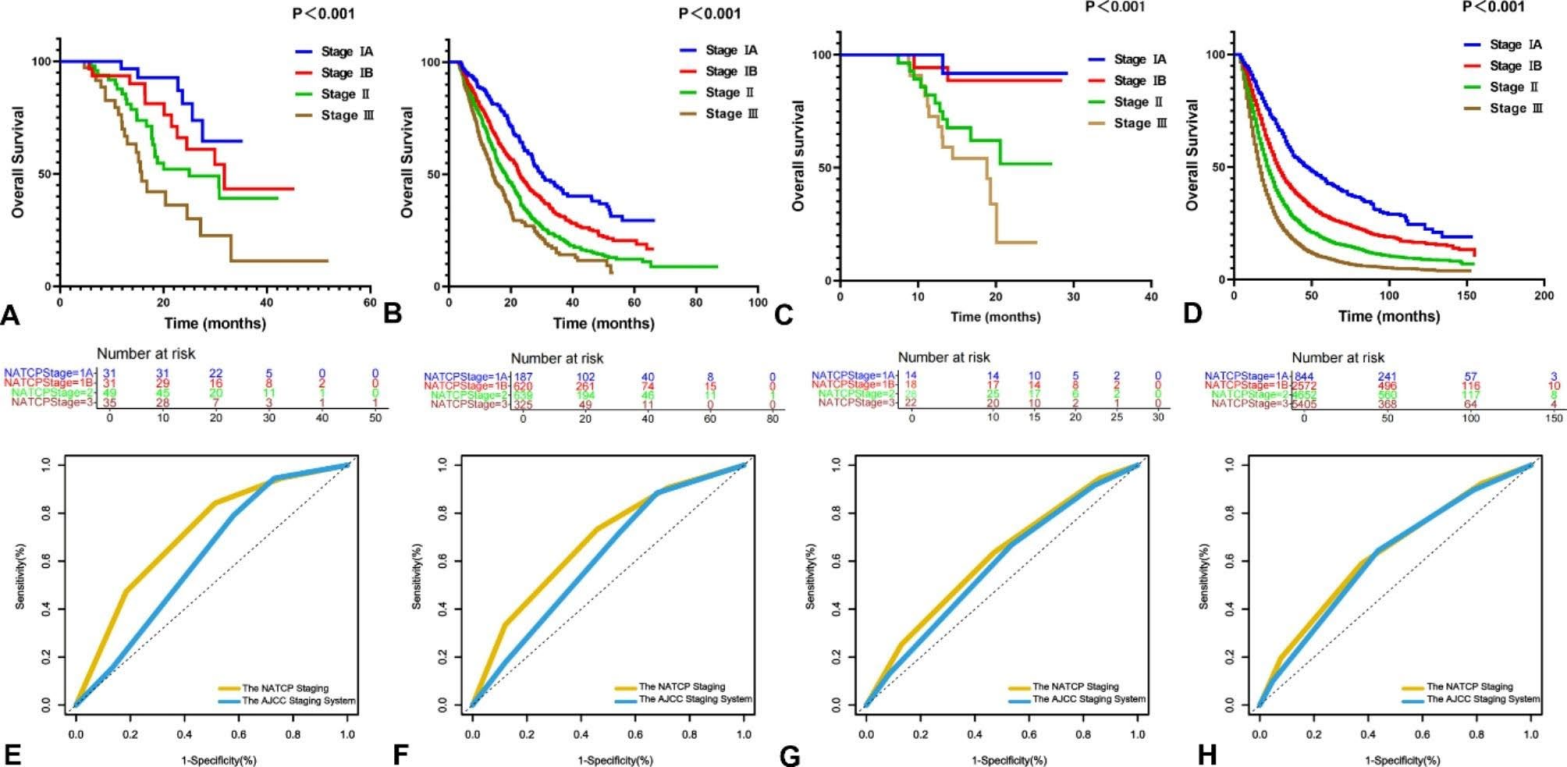
The Kaplan-Meier curves for overall survival of the NAT cohort (**A**), the UFS cohort (**B**), the PNAT cohort (**C**) and the SEER cohort (**D**) using the NATCP staging. The number of patients for NATCP stages IA, IB, II and III was 31, 31, 49, 35 in the NAT cohort (**A**), the number of patients for NATCP stages IA, IB, II and III was 187, 620, 639, 325 in the UFS cohort (**B**), the number of patients for NATCP stages IA, IB, II and III was 14, 18, 28, 22 in the PNAT cohort (**C**), the number of patients for NATCP stages IA, IB, II and III was 844, 2572, 4652, 5405 in the SEER cohort (**D**). Time-dependent area under the receiver operating curves (AUC) demonstrated the predictive ability of the NATCP Staging (yellow) and the AJCC staging system (blue) for 1-yr (**E**), 2-yr (**F**) overall survival in the NAT cohort and for 1-yr (**G**), 2-yr (**H**) overall survival in the UFS cohort. AJCC indicates American Joint Committee on Cancer; NAT, neoadjuvant therapy; PNAT, prospective neoadjuvant therapy; UFS, upfront surgery; SEER, Surveillance, Epidemiology, and End, Results

The follow-up time was 4.7–51.9 months in the NAT cohort. The median OS of the entire cohort was 29.9 (95%CI, 24.4–35.5) months. The median OS for NATCP stages IA, IB, II, and III were not reached, 31.7 months, 25.0 months, and 15.8 months, respectively. The NATCP staging showed good discrimination ability in the entire cohort (Fig. 3A, p<0.001). For the NATCP staging, the Harrell C index for the prediction of 1- and 2-year OS was 0.677 and 0.678, respectively (Supplementary Fig. 1).

The follow-up time was 3.1–87.1 months in the UFS cohort. The median OS of the entire cohort was 20.5 (95%CI, 19.3–21.7) months and for stages IA, IB, II, and III were 30.2 months, 22.8 months, 18.2 months and 14.1 months, respectively. The NATCP staging showed significant discrimination ability in the entire cohort (Fig. 3B, p<0.001).

The survival curves for patients in the PNAT cohort and the SEER cohort were presented in Fig. 3C and D. The median OS of the PNAT cohort could not be statistically analyzed due to the limitations of the sample size and the follow-up time, but a good separation of survival curves by stage was evident (p<0.001). The median OS of the SEER cohort was 21.0 (95%CI, 20.1–21.4) months and for stages IA, IB, II, and III were 47.0 months, 29.0 months, 22.0 months, and 17.0 months, respectively. The good stratification ability of the NATCP staging was also observed in the SEER cohort.

### Comparison with the AJCC staging system

The AJCC staging system had limited prognostic ability in the NAT cohort (p = 0.043), based on no statistical difference in median OS between stages II and III (24.5 vs. 20.4 months, respectively; p = 0.069) (Supplementary Fig. 2). The correlations between the clinicopathologic factors and tumor stage based on AJCC staging and the NATCP staging were detailed demonstrated in Supplementary Table 1.The prognostic performances of the NATCP and AJCC staging systems in the NAT cohort were compared using ROC curves for the prediction of OS at 1 (Fig. 3E) and 2 (Fig. 3F) years. The performance of the NATCP staging was superior to that of the AJCC staging system, with AUC of 0.714 (95%CI, 0.602–0.826) versus 0.614 (95%CI, 0.508–0.720) for 1 year, and AUC of 0.682 (95%CI, 0.580–0.783) versus 0.612 (95%CI, 0.507–0.717) for 2 years, respectively.

The NATCP staging was more accurate for the prediction of 1-year (Fig. 3G) and 2-year (Fig. 3H) OS compared to the AJCC staging system in the UFS cohort, with AUC of 0.612 (95%CI, 0.583–0.641) versus 0.580 (95%CI, 0.552–0.608) at 1 year, and AUC of 0.635 (95%CI, 0.605–0.664) versus 0.620 (95%CI, 0.589–0.649) at 2 years, respectively.

## Discussion

In the present study, we proposed a prognostic staging system that is compatible with NAT and reliably estimates the survival probability of patients receiving NAT or UFS for resected PDAC. Based on the AJCC staging system, the NATCP staging simplified the T group into a binary variable with a cut-off value of 2 cm, as well as added tumor differentiation, and restaged the disease. After these modifications, the prognostic ability was improved compared to the AJCC staging system and was validated in the validation cohorts. The NATCP staging provided accurate prediction of OS for resected PDAC regardless of the use of NAT, which was expected to guide better clinical decision making and postoperative management in the future clinical practice.

To accurately predict prognosis and decide appropriate treatment options, it is vital to stage the disease [20]. The AJCC staging system for PDAC is based on classical pathological parameters: size-based T-stage, lymph node status, and assessment of distant metastases [7]. However, the AJCC staging system was poorly validated in patients receiving NAT. We are now in a new era of neoadjuvant therapy for pancreatic cancer, with treatments that offer the possibility of median survival after resection of even more than 50 months [21]. It is essential to optimize the existing staging system to accommodate new therapy and to help guide the postoperative treatment plans [22]. To demonstrate the good prognostic ability compatibly for patients receiving NAT or UFS for resected PDAC, we simplified T classification, retained the N subgroup, incorporated tumor differentiation and restaged the disease.

We simplified the T group using a cut-off value of 2 cm. Compared to the seventh edition, the eighth edition of the AJCC staging system used only tumor size in the maximum dimension for T1 to T3 [23]. This classification addressed the subjective problem of assessing extrapancreatic extension (since the pancreas has no true envelope) and the fact that more than 90% of tumors were classified as T3. A study of 398 patients who underwent pancreaticoduodenectomy after NAT demonstrated that the eighth edition of the T group better stratified the prognosis of patients compared to the seventh edition. In addition, patients with T1 had better OS than those with T2 or T3. However, there was no statistical difference in OS between T2 and T3 groups [13]. More subgroups of tumors lager than 2 cm did not result in improved prognostic stratification power. Our data also confirmed this finding. A study of 141 patients with BR/LA PDAC who underwent surgical exploration following NAT showed that the T1 group had better OS than T2 or higher group, and tumor size > 2.5 cm in the pathology department were independently associated with decreased OS [24].

The eighth edition of the AJCC staging system classified N group into N0 (no lymph nodes involved), N1 (1–3 positive lymph nodes), and N2 (≥ 4 positive lymph nodes) [7]. The three-category nodal status showed accurate discrimination of survival in resected PDAC patients after NAT [25, 26]. Our results demonstrated that the prognosis of patients with N2 status was significantly worse than that of patients with N0 or N1 status in both the NT and UFS cohorts. However, Macedo et al. observed no difference in OS between N1 and N2 groups for resected PDAC after primary chemotherapy [27]. Fisher et al. reported that a refined N subclassification with node-negative compared to 1, 2, 3, or more lymph nodes involved provided better prognostic discrimination ability [28]. In addition, recent studies suggested that the number of examined lymph nodes and the lymph node ratio (number of positive lymph nodes to the total number of lymph nodes harvested) may be better predictors than lymph node status alone [29–31]. The median number of examined lymph nodes in our institution was 25.

Tumor differentiation was an important prognostic factor of PDAC and should be incorporated into the staging model. In treatment-naïve tumors, the maximum tumor diameter is a good predictor of survival [32]. However, after NAT, the tumor size was often difficult to assess because of treatment-induced fibrosis of the tumor bed and the adjacent parenchyma [33]. Therefore, size alone is an unreliable prognostic factor for PDAC resection patients who received NAT. Furthermore, the biological behavior of pancreatic cancer was more unpredictable than other solid tumors. In a study of nearly 59,000 patients from the SEER database, only 0.3% of patients had tumors size of ≤ 0.5 cm or less, but almost 31% of patients had distant metastasis at the time of diagnosis [34]. The tumors with small size may have poor biological behaviors, while large tumors may have similar biological behaviors, suggesting the need to consider additional pathological indicators reflecting the biological characteristics of the tumor for staging. Tumor differentiation indicates the morphological and functional similarities between malignant cells and tissue of origin. Epithelial malignancies can range from well/moderately differentiated tumors resembling the tissue of origin to poorly differentiated/undifferentiated tumors with indistinguishable tissue origin [14]. A recent high-quality study showed that tumor differentiation, a morphological parameter, is most likely associated with the molecular subtypes of PDAC (Classical/Basal), which may also explain the strong correlation between tumor differentiation with prognosis [35, 36]. The molecular subtypes of pancreatic cancer are related to the sensitivity of different chemotherapy regimens, and the potential biological role of tumor differentiation will be gradually revealed with further morphological studies in the future. Tumor differentiation is a significant independent prognostic factor for OS in resected PDAC without NAT. The addition of tumor differentiation to the TNM staging system improved survival discrimination ability compared to the AJCC system [14, 37−41]. Two previous studies reported that tumor differentiation is also an independent prognostic factor for OS in resected PDAC after NAT [13, 26]. Our study supported the findings and performed multivariate analysis that showed that tumor differentiation was the strongest predictive factor for OS (p = 0.003). To our knowledge, the NATCP staging is the first attempt to add tumor differentiation to the pathological staging of patients who underwent resection after NAT.

To increase the accuracy of the staging system and to facilitate good separation of the survival curves, we regrouped the substages in the NATCP staging according to the survival duration. In short, the poorly differentiated or undifferentiated tumors from the previous stage were brought into the next stage. In particular, the curves for stage II and III patients in the NAT cohort are not well-separated in the current AJCC staging system. Similar results were obtained in a study of 216 patients who underwent resection for PDAC after NAT [42]. However, after restaging, the survival curves using the NATCP staging were sufficiently separated between stages.

In multivariate analysis, tumor differentiation and the number of positive lymph nodes were independent prognostic factors for OS in the NAT cohort. Strict R0 resection was reported to improve the OS in patients who underwent UFS or NAT for resected PDAC [20, 43]. However, R0 resection was not an independent predictor of survival in the present (p = 0.385), which may be related to the fact that the vast majority of patients in the NAT cohort received postoperative adjuvant therapy. A cohort study of 501 patients undergoing pancreaticoduodenectomy showed that adjuvant therapy improved survival and equalized survival curves for patients with positive and negative margins, regardless of resection status [44].

The strategy of simplifying the T group and adding tumor differentiation to refine the TNM staging system in patients receiving NAT has been used for other types of digestive tract tumors. Based on the national SEER database, Yuan et al. [45] proposed that in esophageal cancer with NAT, combining the T1 and T2 subgroups, and adding tumor differentiation, can significantly improve the performance of the TNM staging system.

There were some limitations in this study. First, because of the retrospective study data and the limited sample size, the influence of potential confounding factors cannot be excluded. Second, mixing all patients into one cohort to design the staging system seems more appropriate for the topic of compatibility, however, the overall analysis will dilute the impact of neoadjuvant treatment due to unbalanced sample size between the NAT and UFS cohorts, resulting in staging that is not truly compatible. Finally, considering the compatibility of the staging system, NAT-specific clinicopathological factors, such as tumor regression grade (TRG) and RECIST1.1, were not included in the analysis. Further research is needed to overcome these limitations.

## Conclusion

In this study, we proposed a NATCP staging to improve the compatibility of the AJCC staging system in predicting survival in PDAC patients who underwent resection after NAT. The median OS for NATCP stages IA, IB, II and III were not reached, 31.7 months, 25.0 months, 15.8 months in the NAT cohort and 30.1 months, 22.8 months, 18.3 months, 14.1 months in the UFS cohort. The NATCP staging provided better prognostic ability than the current AJCC staging system for resected PDAC regardless of the use of NAT and may facilitate clinical decision-making based on accurate prediction of OS of patients.

### Electronic supplementary material

Below is the link to the electronic supplementary material.

Supplementary Material 1

Supplementary Material 2

Supplementary Material 3

## Data Availability

The datasets generated during and/or analyzed during the current study are available from the corresponding author on reasonable request.
