# Supplementary figures and images for: A neoadjuvant therapy compatible prognostic staging for resected pancreatic ductal adenocarcinoma
Source: BMC Cancer. 2023 Aug 23;23:790. doi: 10.1186/s12885-023-11181-x (PMC10463422; doi:10.1186/s12885-023-11181-x)

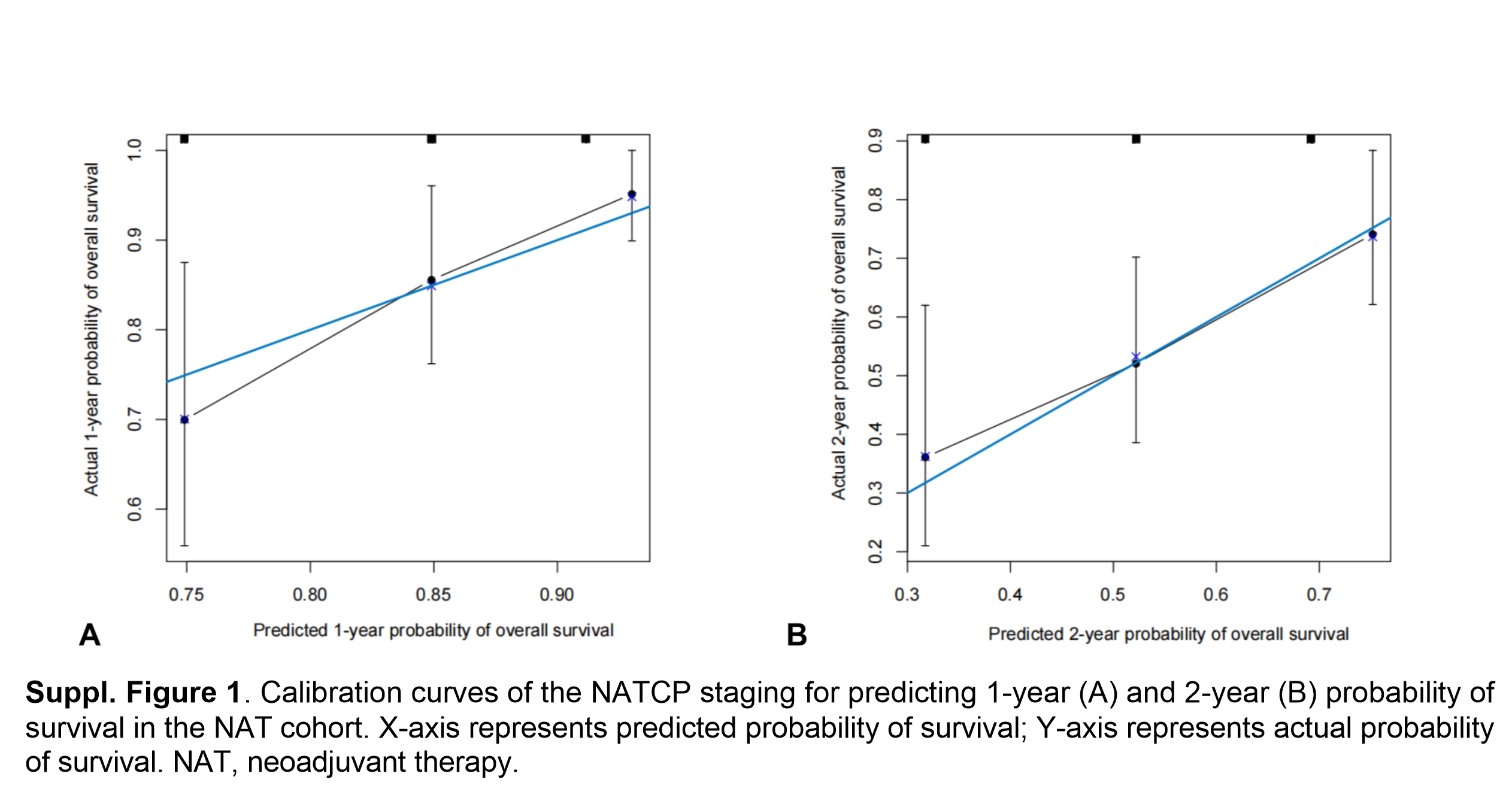

Supplement: Supplementary file 2 — Supplementary Material 2 [file 12885_2023_11181_MOESM2_ESM.tif]

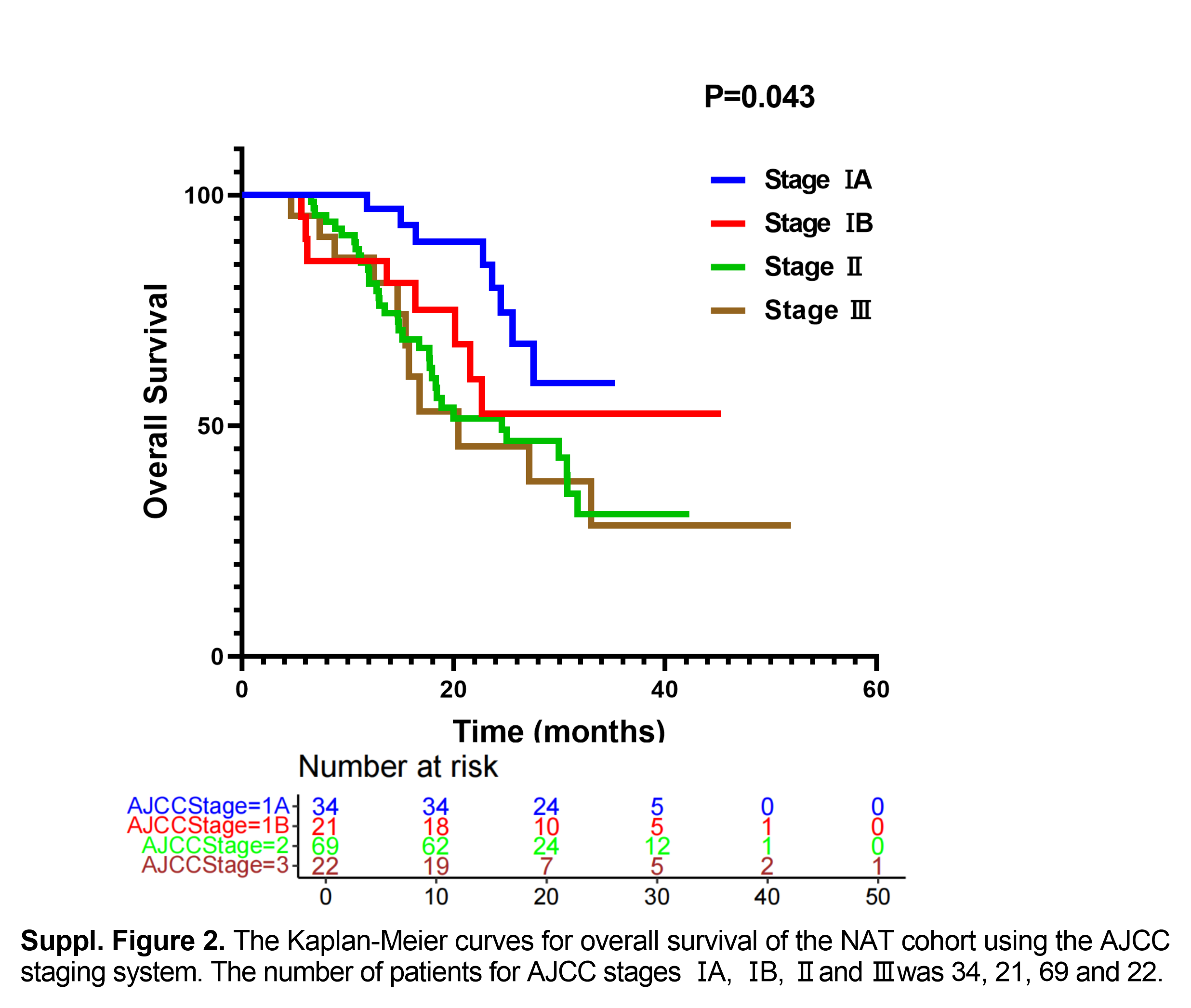

Supplement: Supplementary file 3 — Supplementary Material 3 [file 12885_2023_11181_MOESM3_ESM.tif]
